# Polyphenol blend enhances zootechnical performance, improves meat quality, and reduces the severity of wooden breast in broiler chickens

**DOI:** 10.3389/fvets.2025.1584897

**Published:** 2025-04-11

**Authors:** Vivian Aparecida Rios de Castilho Heiss, Maria Fernanda de Castro Burbarelli, Bruna Barreto Przybulinski, Letícia Cuer Garcia, João Ricardo Rodrigues Ferreira Vieira, Rodrigo Garófallo Garcia, Fabiana Ribeiro Caldara, Elizabeth Santin, Claudia Andrea Lima Cardoso, Claudia Marie Komiyama

**Affiliations:** ^1^Faculty of Agricultural Sciences, Federal University of Grande Dourados, Dourados, Mato Grosso do Sul, Brazil; ^2^Kupono Assessoria e Consultoria, Curitiba, Paraná, Brazil; ^3^Mato Grosso do Sul State University, Dourados, Mato Grosso do Sul, Brazil

**Keywords:** condensed tannins, flavonoids, hydrolyzable tannins, meat quality, oxidative stress, pectoral myopathies

## Abstract

This study investigated the effects of a commercial polyphenol blend on broiler performance, meat quality, carcass traits, and the incidence of pectoral myopathies. Broilers (1–42 days old) were allocated to four treatments: T1 (control, basal diet), T2 (250 g/ton polyphenol blend), T3 (500 g/ton), and T4 (1,000 g/ton), with eight replicates of 40 birds each. All diets were corn-soy based, isonutritional, and formulated to meet age-specific nutritional requirements. Parameters assessed at 21, 28, 35, and 42 days included antioxidant potential, growth performance, myopathy incidence, carcass yield, allometric growth, muscle morphometry, meat quality, and lipid profile. Optimal performance was observed at a supplementation level of 514 g/ton of polyphenols. While carcass yield remained unaffected, birds fed 500 g/ton exhibited delayed breast growth relative to other body parts, suggesting modulated allometric growth. Polyphenol supplementation reduced breast muscle fiber size, increased fiber density, and lowered the severity of wooden breast without influencing the incidence of white striping. Improved meat tenderness was evident through reduced cooking weight loss and enhanced shear force. Antioxidant status improved in plasma, muscle, and liver tissues, and the muscle lipid profile was favorably altered. In conclusion, the polyphenol blend enhanced broiler zootechnical performance, alleviated wooden breast severity, and improved meat quality and tenderness.

## 1 Introduction

Broiler chicken myopathies, such as wooden breast and white striping, lead to the condemnation of carcasses and prime commercial cuts during slaughter, resulting in significant economic losses for the poultry industry. Wooden breast is characterized by localized or diffuse areas of hardness in the pectoral muscle, sometimes accompanied by viscous material and petechiae (1). White striping manifests as white lines running parallel to the muscle fibers, which are prominently visible in raw meat, primarily affecting the breast and thigh muscles (2, 3).

The myodegeneration associated with white striping and wooden breast is well documented to reduce total protein content and alter protein profiles (4). Histologically, these myopathies are characterized by myodegeneration, necrosis, fibrosis, lipidosis, and regenerative changes in the pectoralis major muscle (5).

These myopathies are strongly correlated with the fast growth rates of broiler chickens (6) and genetic selection for increased breast size, which compromise blood and oxygen supply to the muscle tissue. This leads to severe hypoxia (7–9) and cellular damage, as the removal of metabolic waste products is also impaired (10–12). Evidence suggests that muscle tissue attempts to counteract hypoxia by synthesizing nitric oxide to increase blood flow, but this response can exacerbate and accelerate oxidative stress. This pro-oxidative environment contributes to tissue inflammation and further myodegeneration (7). Although the link between pectoral myopathies and oxidative stress is not yet fully elucidated, studies have reported elevated levels of oxidative markers in chickens affected by both white striping and wooden breast (13, 14). Animals with higher growth rates and accelerated metabolism may be particularly susceptible to oxidative stress due to increased production of reactive oxygen species (ROS) (15).

This study hypothesizes that nutritional strategies may reduce oxidative stress and mitigate myopathy severity. Polyphenols, a diverse group of phytogenic substances found in plant components such as leaves, seeds, flowers, and fruits (16), have demonstrated antioxidant, anti-inflammatory, and anti-mutagenic properties (17, 18). Polyphenols can be categorized into two main groups: non-flavonoids, including lignans, stilbenes, and phenolic acids (e.g., tannins), and flavonoids, such as flavones, flavonols, flavanols, flavanones, isoflavonoids, and anthocyanins (19).

The antioxidant mechanism of polyphenols involves scavenging free radicals or stimulating the synthesis of ROS-removing enzymes, including superoxide dismutase, catalase, and glutathione peroxidase (16, 20, 21). Consequently, polyphenols are promising candidates for poultry nutrition to mitigate myopathies and enhance meat quality. Previous research has shown that polyphenols positively influence the performance and intestinal health of production animals (16, 21, 22).

Therefore, this study aimed to evaluate the effects of different levels of a commercial polyphenol blend in broiler chicken diets on zootechnical performance, the allometric growth of body parts, the incidence of white striping and wooden breast myopathies, meat quality parameters, muscle morphometry, antioxidant potential, and lipid profile.

## 2 Materials and methods

### 2.1 Birds and housing

This study was approved by the Ethics Committee on the Use of Animals (CEUA/UFGD) under protocol number 02/2022. The experiment was conducted in the poultry house of the School of Agricultural Sciences at the Federal University of Grande Dourados (FCA/UFGD). The facility was equipped with a negative pressure system, pendulum drinkers, tubular feeders, and 250 W infrared lamps. For the first week, the light program provided 23 h of continuous light, which was gradually reduced over 11 days. Thereafter, birds were exposed to 18 h of intermittent light per day at an intensity of 22 lumens. Temperature and humidity were monitored every 15 min using the RC-51 data logger (Elitech Brasil).

The study included 1,280 one-day-old male Ross AP91® chicks raised at a density of 14 birds/m^2^. Birds were allocated to a completely randomized design comprising four experimental diets with eight replicates of 40 birds each: T1 (control, basal diet), T2 (250 g/ton of a commercial polyphenol blend, Silvateam ATX), T3 (500 g/ton of ATX), and T4 (1,000 g/ton of ATX).

The polyphenol blend (Silvafeed ATX®) was a powdered additive rich in polyphenols, flavonoids, and tannins, produced by SilvaTeam (San Michele Mondovì CN, Italy). Diets were formulated using corn and soybean meal to meet nutritional requirements based on Rostagno et al. (23). Feed was provided *ad libitum* in four phases according to age: pre-starter (1–7 days), starter (8–21 days), grower (22–35 days), and finisher (36–42 days) (Table 1). Management practices followed the guidelines specified in the Ross manual.

**Table 1:** Composition of experimental diets according to the productive phase in meeting the nutritional requirements for male broilers in 100 kg of feed.

Ingredients	Pre-starter	Starter	Growth	Final
Corn	42.859	46.260	53.138	62.820
Soybean meal	46.214	42.750	35.593	27.675
Soy oil	5.096	5.863	6.182	5.003
Dicalcium phosphate	2.332	2.133	1.822	1.471
Limestone	0.763	0.721	0.557	0.425
salt	0.489	0.473	0.449	0.421
DL-Methionine	0.351	0.327	0.286	0.229
L-Lysine	0.106	0.224	0.179	0.217
L-Threonine	0.060	0.059	0.064	0.059
Choline chloride	0.080	0.080	0.080	0.080
Zinc Bacitracin	0.050	0.050	0.050	-
Vitamin premix^1^	0.030	0.030	0.030	0.030
Mineral premix^2^	0.030	0.030	0.030	0.030
Inert	1.000	1.000	1.000	1.000
Total	100.000	100.000	100.000	100.000
Requirements %				
ME, kcal/kg	3.000	3.100	3.200	3.250
Crude protein	25.010	23.750	21.040	18.290
Calcium	1.029	0.911	0.758	0.606
Available *p*	0.553	0.525	0.458	0.383
Sodium	0.210	0.210	0.201	0.191
Lysine	1.369	1.366	1.161	1.008
Methionine	0.667	0.627	0.558	0.473
Methionine + Cystine	1.011	0.955	0.856	0.742
Threonine	0.905	0.852	0.763	0.661

^1^Vitamin supplement inclusion levels per 100 kg of feed: Folic acid (min.) 225,000 mg; Pantothenic acid (min.) 3.00 g; Biotin (min.) 24.00 mg; Niacin (min.) 12,000 g; Vitamin A (min.) 2,400,000.00 IU; Vitamin B1 (min.) 900.00 mg; Vitamin B12 (min.) 5,400.00 μg; Vitamin B2 (min.) 1,800.00 mg; Vitamin B6 (min.) 975.00 mg; Vitamin D3 (min.) 750,000.00 IU; Vitamin E (min.) 4,500.00 IU; Vitamin K3 (min.) 750.00 mg. ^2^Mineral supplement per 100 kg of feed: Copper (min.) 1,890.00 mg; Iron (min.) 15.75 g; Iodine (min.) 378.00 mg; Manganese (min.) 21.00 g; Selenium (min.) 90.00 mg; Zinc (min.) 18.90 g.

### 2.2 Zootechnical performance

Zootechnical performance was evaluated during the 1–7, 1–21, 1–35, and 1–42 days periods, with parameters including feed intake (FI), weight gain (WG), feed conversion ratio (FCR), and the European Broiler Production Efficiency Index (EBI). All birds were weighed weekly to determine WG, while feed supplied and leftover feed were recorded to calculate FI. The FCR was derived from the ratio of FI to WG. The EBI was calculated as:


$$EBI=\frac{\mathrm{Average}\;\mathrm{gain}\;\mathrm{day}\;\left(g\right)\;x\%\mathrm{viability}}{\mathrm{Feed}\;\mathrm{convertion}\;\mathrm{ratio}}$$

### 2.3 Carcass and cut yield and calculation of the allometric coefficient

All birds were weighed at 21, 28, 35, and 42 days of age. Two birds per replicate (16 birds per treatment), representing the average pen weight (±5%), were selected to evaluate carcass and cut yield, as well as meat quality and pectoral myopathy incidence (white striping and wooden breast).

The birds were fasted for 8 h, slaughtered via cervical dislocation, and exsanguinated by severing the jugular veins and carotid arteries. Carcasses underwent scalding, plucking, evisceration, pre-cooling (10–18°C for 12 min), cooling (0–2°C for 18 min), and dripping (5 min). Carcasses were weighed post-evisceration (hot), post-dripping (chilled), and then processed for cuts.

Yields (%) were calculated based on live weight for hot and chilled carcasses (excluding feet and head) and based on chilled carcass weight for cuts (bone-in breast, boneless skinless breast, thighs and drumsticks, wings, and back).

The relative growth or allometric coefficient of commercial cuts and chilled carcass weight was determined using the methodology described by Castilho et al. (24).

### 2.4 Classification of myopathies and meat quality analysis

Boneless breast filets were classified for myopathy severity at 21, 28, 35, and 42 days of age. White striping was scored using the four-point scale established by Kuttappan et al. (25), while wooden breast was classified by manual palpation, following the guidelines of Sihvo et al. (12). A trained evaluator conducted the classifications.

Filets were stored at 5°C for 24 h before meat quality analyses, which included dimensions (length, width, thickness) (26), pH (after 24 h), objective color (27), water holding capacity (28), drip loss (29) weight loss from thawing/cooking (30), and shear force (31). Sampling for each filet was standardized according to Castilho et al. (24).

### 2.5 Muscle morphometry

Fragments of the pectoralis major muscle were collected from one bird per replicate (eight birds per treatment) at 21, 28, 35, and 42 days of age for morphometric analysis. Samples (~200 mg) were cut into parallelepiped shapes, oriented parallel to the muscle fibers, and had their surfaces removed. Samples were immediately frozen in liquid nitrogen and stored at −80°C (96).

Frozen samples were sectioned using a Lupetec® CM 2850 cryostat microtome at −20°C. Samples were fixed onto the cryostat’s metallic support using a special resin. Histological sections were obtained, considering two slides per bird and 10 sections per slide, with a thickness of 10 μm. The sections were then subjected to Hematoxylin–Eosin (HE) staining to analyze the general morphometry of muscle fibers.

A total of 10 photomicrographs per sample were obtained using a Precision® P207BI binocular microscope equipped with a BEL Engineering® Eurekam 1.3 camera, connected to a computer, and analyzed with the Image Pro-Plus® software. The images were captured under 10x objective magnification, combined with a 10x ocular lens, resulting in a total magnification of 100x, allowing for a detailed evaluation of the muscle fiber structure. The analyses included fiber count per field, representing the number of fibers in the cross-sectional area; fiber diameter (μm), which corresponds to the mean measurement of the largest diameter of each muscle fiber per field; fiber area (μm^2^), represented by the mean measurement of each muscle fiber’s area per field; and the fiber area per field (%), which represents the percentage of the cross-sectional area occupied by muscle fibers, excluding free spaces, as described by Castilho et al. (32).

### 2.6 Antioxidant activity

Plasma, 3 g of liver, and *Pectoralis major* muscle samples were collected from five birds per treatment at 21 and 42 days of age. Plasma was obtained by collecting blood samples from the ulnar vein in tubes containing an anticoagulant, followed by centrifugation to separate the blood components immediately after collection. The samples were centrifuged at 10,000 g for 15 min at 4°C, allowing the separation of plasma, which was carefully collected and stored at an appropriate temperature (−80°C) until analysis. Liver and muscle samples were stored at −80°C and subsequently lyophilized. To evaluate antioxidant potential, liver and muscle samples were freeze-dried, pulverized, and extracted with 5 mL of hexane for 30 min using ultrasound. Following extraction, the hexane phase was filtered and evaporated under a fume hood. The remaining solid residue underwent further sequential extraction using ethyl acetate and ethanol under the same conditions. The resulting filtrates were combined, dried, and solubilized in ethanol to form a single extract. This extract was analyzed for antioxidant activity using the DPPH (2,2-diphenyl-1-picrylhydrazyl) assay. Plasma samples were analyzed directly without prior extraction.

To assess the DPPH inhibition capacity, 3 mL of 0.1 mM DPPH solution was mixed with triplicate sample aliquots at varying concentrations. Absorbance at 517 nm was measured using a spectrophotometer. The percentage of inhibition was calculated following the method described by Kumaran (33).

### 2.7 Determination of fatty acid profile

The lipid fraction of Pectoralis major muscle samples from 42-day-old birds was extracted following the method of Bligh and Dyer (34). The lipid extract was weighed, and 60 mg of it was methylated according to Maia and Rodriguez-Amaya (35) for gas chromatography analysis.

Fatty acid methyl ester analysis was conducted using a gas chromatograph (Thermo) equipped with a flame ionization detector, a “split/splitless” injector, and a fused silica capillary column with polyethylene glycol as the stationary phase (DB-Wax, 30 m × 0.25 mm, J&W Scientific). Chromatographic conditions were as follows: injector temperature at 250°C; column temperature at 180°C for 20 min, followed by a programmed increase of 2°C/min to 220°C; detector temperature at 260°C; hydrogen carrier gas at a flow rate of 1.0 mL/min; nitrogen make-up gas at 20 mL/min; and injection volume of 1 μL.

Fatty acid identification was based on retention time comparisons with methyl ester standards (Sigma-Aldrich). Quantification was performed via area normalization, and results were expressed as the percentage of each fatty acid relative to the total fatty acid area.

### 2.8 Serum biochemical profile

Blood samples (5 mL/bird) were collected from the ulnar vein of one bird per replicate (eight birds per treatment) using heparinized syringes at 42 days of age. Samples were collected at the experimental poultry house, ensuring that they were obtained under standardized conditions. Samples were centrifuged at 4,000 rpm for 10 min to separate the serum, which was subsequently stored at −80°C until analysis.

To assess clinical markers of muscle damage, serum alanine aminotransferase (ALT), aspartate aminotransferase (AST), and lactate levels were evaluated. ALT and AST were analyzed using a spectrophotometer (BioPlus 200) as per the manufacturer’s instructions (GoldAnalisa®). Lactate levels were measured with the Accutrend Plus Roche® device and BM-Lactate® test strips.

### 2.9 Statistical analysis

The normality of residuals and homogeneity of variances were assessed using the Shapiro–Wilk and Levene tests, respectively. Subsequently, the data were subjected to analysis of variance (ANOVA) using the SAS MIXED procedure (SAS, version 9.4; SAS Institute Inc., Cary, NC, United States). For performance and live weight data, the mathematical model included covariates to adjust for initial conditions. When the statistical model was significant, polynomial regression analysis was used to estimate the effects of polyphenol blend inclusion levels.

Regression models were evaluated based on the significance of parameters, the coefficient of determination (R^2^), and the alignment of estimated levels with biological plausibility. Coefficients of determination were classified as high (0.70–1.00), moderate (0.40–0.69), or low (0.00–0.39). Statistical significance was set at a 5% level (*p* < 0.05).

Since the results for myopathy assessments were not normally distributed, generalized linear models (GLMs) were applied following the methodology of Nelder and Wedderburn (36) using the SAS GLIMMIX procedure (97). Data distributions were modeled as gamma (GAMMA) if residuals exhibited exponential behavior. Regression coefficients and intercepts were obtained using the SOLUTION option of the GLIMMIX procedure. To compare means, “lsmeans” were calculated using the “inverse link” function, with adjustments made using the pdiff ilink lines statement in the GLIMMIX procedure.

The study of relative growth of body cuts was analyzed using the power equation Y = aXbY = a Xb, which was transformed logarithmically into a linear model, lnY = lna + b lnX + lnei (24, 37, 38).

## 3 Results

### 3.1 Environmental conditions and zootechnical performance, carcass and cut yield, and allometry

During the study, temperature and humidity were within the expected range for the age of the animals (Table 2). However, from 11 to 42 days, daily temperature variations ranged from 7 to 10°C.

**Table 2:** Temperature and relative humidity.

	Temperature (°C)				Relative humidity (%)			
Week	Desired	Min	Max	Average	Desired	Min	Max	Average
1	32	29	33	31	50–70	59	72	65
2	29–23	23	29	26	50–70	64	72	68
3	23	20	28	24	50–70	55	64	59
4	23	21	27	24	50–70	66	74	70
5	23–22	21	27	24	50–70	66	72	69
6	22–18	21	27	24	50–70	59	65	62

**Figure 1:**
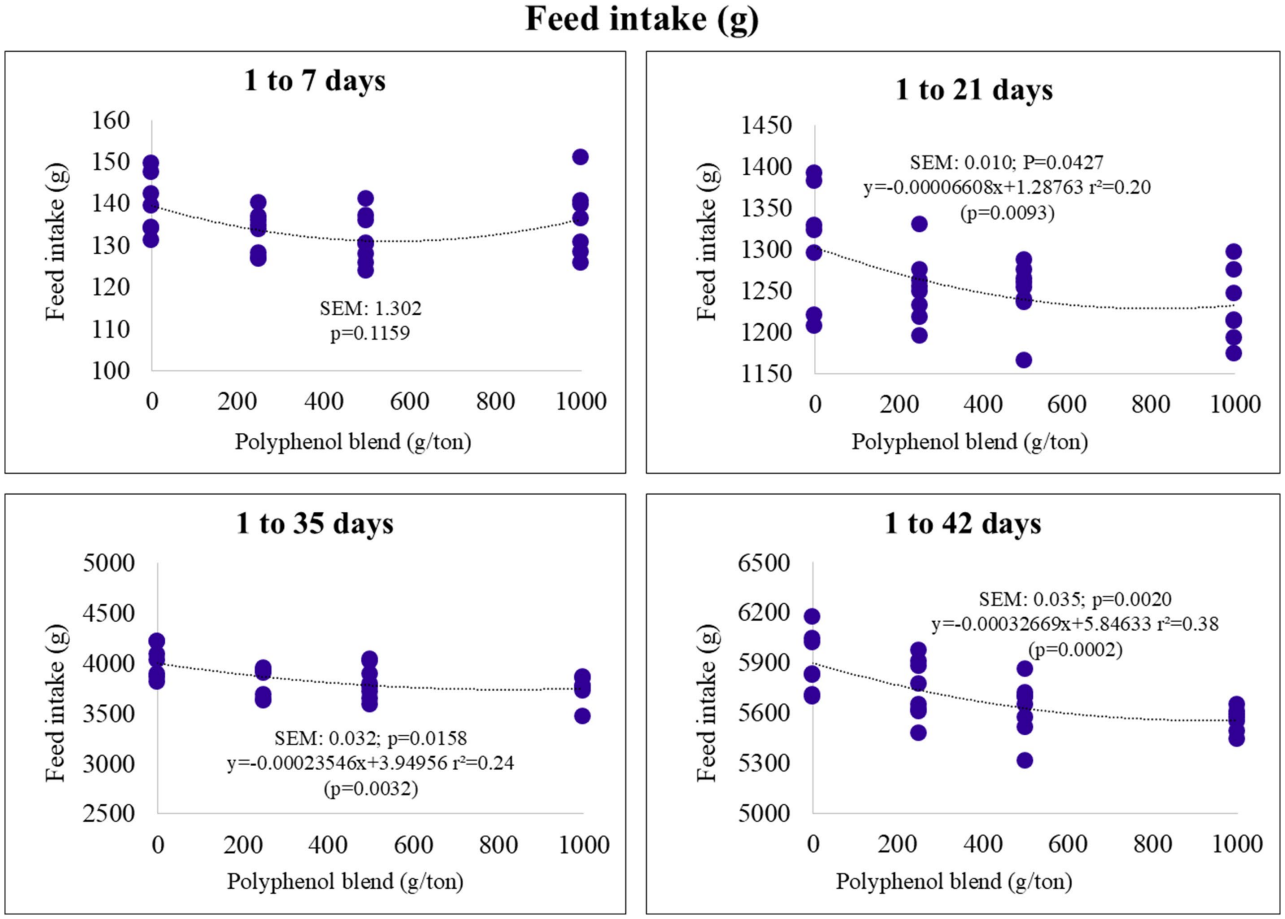
Feed intake (g) of broiler chickens fed on diets containing different levels of polyphenol blend.

Feed intake demonstrated a linear decrease over the cumulative periods of 1–21, 1–35, and 1–42 days of age. Specifically, higher levels of polyphenol blend in the diet were associated with lower feed consumption (Figure 1).

**Figure 2:**
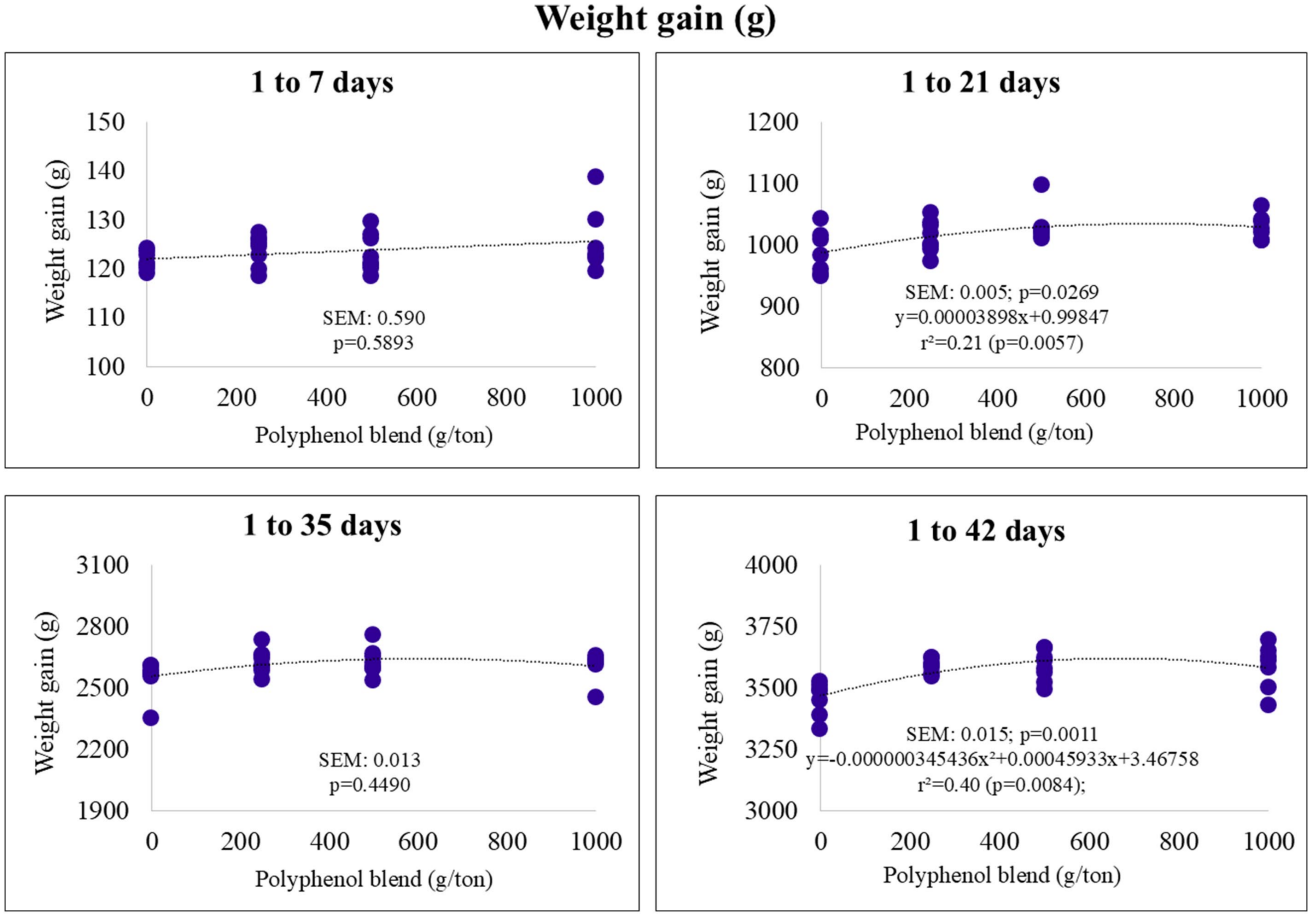
Weight gain (g) of broiler chickens fed on diets containing different levels of polyphenol blend.

In the starter phase (1–21 days), weight gain exhibited a linear increase with higher polyphenol inclusion. In the final phase (1–42 days), it followed a quadratic trend, with an estimated maximum of 3.620 kg at 664 g/ton of polyphenol inclusion (Figure 2).

**Figure 3:**
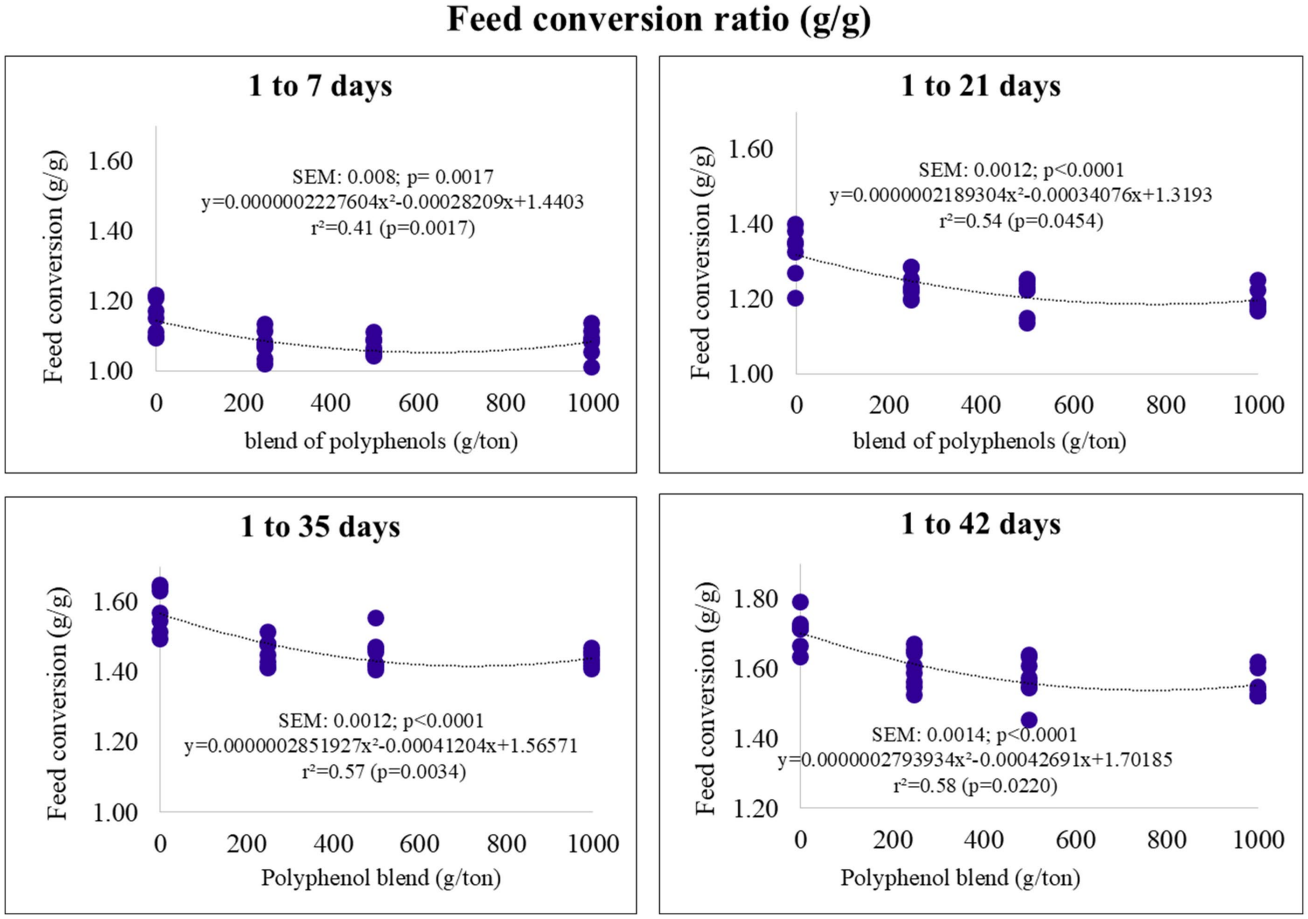
Feed conversion ratio (FCR) of broiler chickens fed on diets containing different levels of polyphenol blend.

The feed conversion ratio (FCR) exhibited a quadratic response, with minimum points observed at polyphenol blend inclusion levels of 633, 778, 722, and 763 g/ton for the pre-starter, starter, growth, and final phases, respectively. The corresponding FCR values were estimated at 1.09, 1.18, 1.41, and 1.53 (Figure 3).

**Figure 4:**
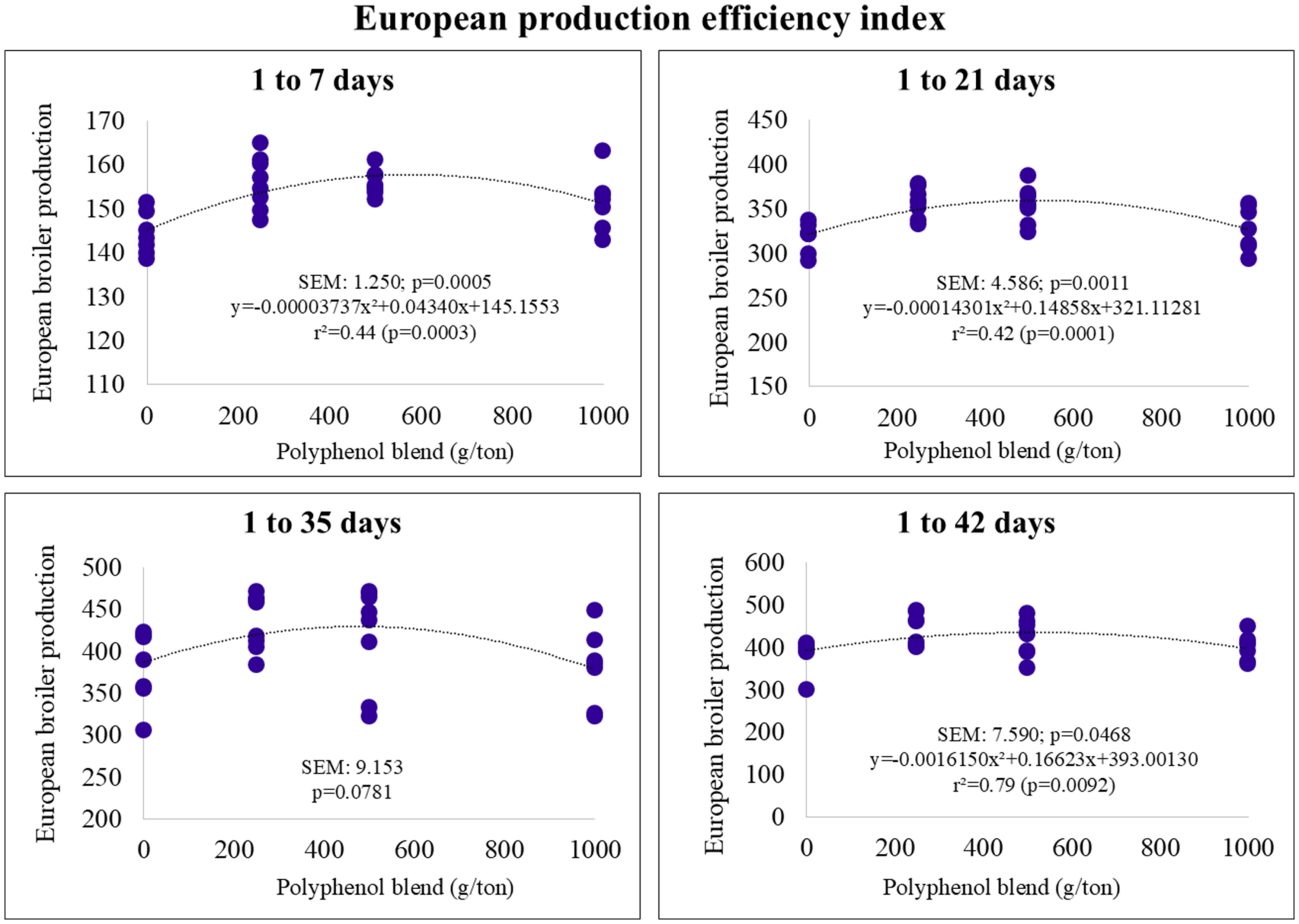
European Production efficiency index of broiler chickens fed on diets containing different levels of polyphenol blend.

The European Production Efficiency Index (EBI) also followed a quadratic trend. The maximum estimated inclusion levels for EBI were 580, 519, and 514 g/ton for the pre-starter, starter, and final phases, respectively (Figure 4). The optimal EBI values were estimated at 157 (pre-starter), 359 (starter), and 477 (final).

Carcass yield and the yield of bone-in and boneless cuts from broilers at 21, 28, 35, and 42 days of age were not significantly influenced by the inclusion of the polyphenol blend, except for back yield at 42 days (Table 3). Back weight showed a linear decrease, but the low coefficient of determination indicated that the regression equation poorly explained the data variation.

**Table 3:** Carcass and cut yield of broiler chickens fed on different levels of polyphenol blend at 42 days of age.

	Blend of polyphenols inclusion (g/ton)				SEM^1^	*p* ^2^
	0	250	500	1,000		
Live weight (kg)	3.444	3.346	3.350	3.321	0.018	0.0909
Carcass yield (%)						
Hot carcass	81.154	81.144	81.985	80.771	0.226	0.2750
Chilled carcass	81.801	81.930	82.760	81.579	0.211	0.2071
Breast with bone	42.656	47.726	42.448	42.435	0.269	0.9748
Legs with bones	26.127	26.416	26.403	26.603	0.140	0.7253
Boneless breast	35.435	35.487	35.371	35.453	0.253	0.9988
Boneless legs	18.440	18.788	18.623	18.333	0.133	0.6459
Wings	9.914	9.265	9.139	9.368	0.054	0.5035
Back	17.648	16.957	17.193	16.639	0.180	0.2731

^1^Standard error of the mean; ^2^Results were statistically significant at the level of *p* < 0.05; Back: y = −0.00003547x + 0.48822 r^2^ = 0.09 (*p* = 0.0164).

For allometric growth coefficients, the control diet (without polyphenol blend inclusion) revealed isogonic growth (b = 1) for the back, indicating similar growth rates to the overall body (Table 4). In contrast, the breast, legs, and wings (except for the inclusion of 250 g/ton) exhibited heterogonic growth (b ≠ 1). The breast and wings displayed early growth (b < 1), while the legs showed late growth (b > 1) in the control treatment.

**Table 4:** Allometric coefficient of broiler chickens slaughtered at 42 days of age fed on diets containing different levels of polyphenol blend.

Cut	Equation	T test (Ho:b = 1)	b ± SD	R^2^
Control				
Breast	lnbreast = 0.87653lnPCRajlog-0.73684	0.0036	0.87653 ± 0.243	0.5192
Legs	lnlegs = 1.35947lnPCRajlog-1.69675	0.0001	1.35947 ± 0.240	0.7268
Wings	lnwings = 0.61711lnPCRajlog-1.98321	0.0194	0.61777 ± 0.228	0.3777
Back	lnback = 0.95973lnPCRajlog-1.68313	0.0649	0.95973 ± 0.472	0.2559
250 g/ton				
Breast	lnbreast = 0.81453lnPCRajlog-0.67101	0.0060	0.81453 ± 0.251	0.4274
Legs	lnlegs = 0.49858lnPCRajlog-0.83058	0.0083	0.48958 ± 0.162	0.4027
Wings	lnwings = 0.28949lnPCRajlog-1.66604	0.1111	0.28949 ± 0.170	0.1712
Back	lnback = 2.28583lnPCRajlog-3.04390	0.0009	2.28583 ± 0.543	0.5582
500 g/ton				
Breast	lnbreast = 1.31950 lnPCRajlog-1.17563	0.0014	1.31950 ± 0.332	0.5295
Legs	lnlegs = 0.65310 lnPCRajlog-0.97504	0.0409	0.65310 ± 0.290	0.2658
Wings	lnwings = 1.09953 lnPCRajlog-2.48829	0.0041	1.09953 ± 0.321	0.4555
Back	lnback = 0.67209 lnPCRajlog-1.42540	0.2766	0.67209 ± 0.593	0.0839
1,000 g/ton				
Breast	lnbreast = 0.69884 lnPCRajlog-0.53317	0.0045	0.69884 ± 0.200	0.5030
Legs	lnlegs = 0.37654 lnPCRajlog-0.70117	0.0342	0.37654 ± 0.157	0.3224
Wings	lnwings = 0.55864 lnPCRajlog-1.92507	0.0376	0.55864 ± 0.239	0.3128
Back	lnback = 5.05244 lnPCRajlog-5.87934	<0.0001	5.05244 ± 0.628	0.8435

ln, Logarithmic transformation for a linear equation; PCRaj, Adjusted chilled carcass weight; t-test, indicates the acceptance or rejection of the H0 hypothesis (*p* < 0.05); R^2^, the value of the equation.

The inclusion of 250 g/ton of the polyphenol blend resulted in early growth for the breast and legs (negative heterogonic growth), late growth for the back (positive heterogonic growth), and isogonic growth for the wings (b = 1). At 500 g/ton, breasts and wings showed late growth, legs showed early growth, and back exhibited isogonic growth. At 1,000 g/ton, early growth was observed for the breast, legs, and wings, while the back showed late growth.

### 3.2 Quantification of myopathies, meat quality, and muscle morphometry

**Figure 5:**
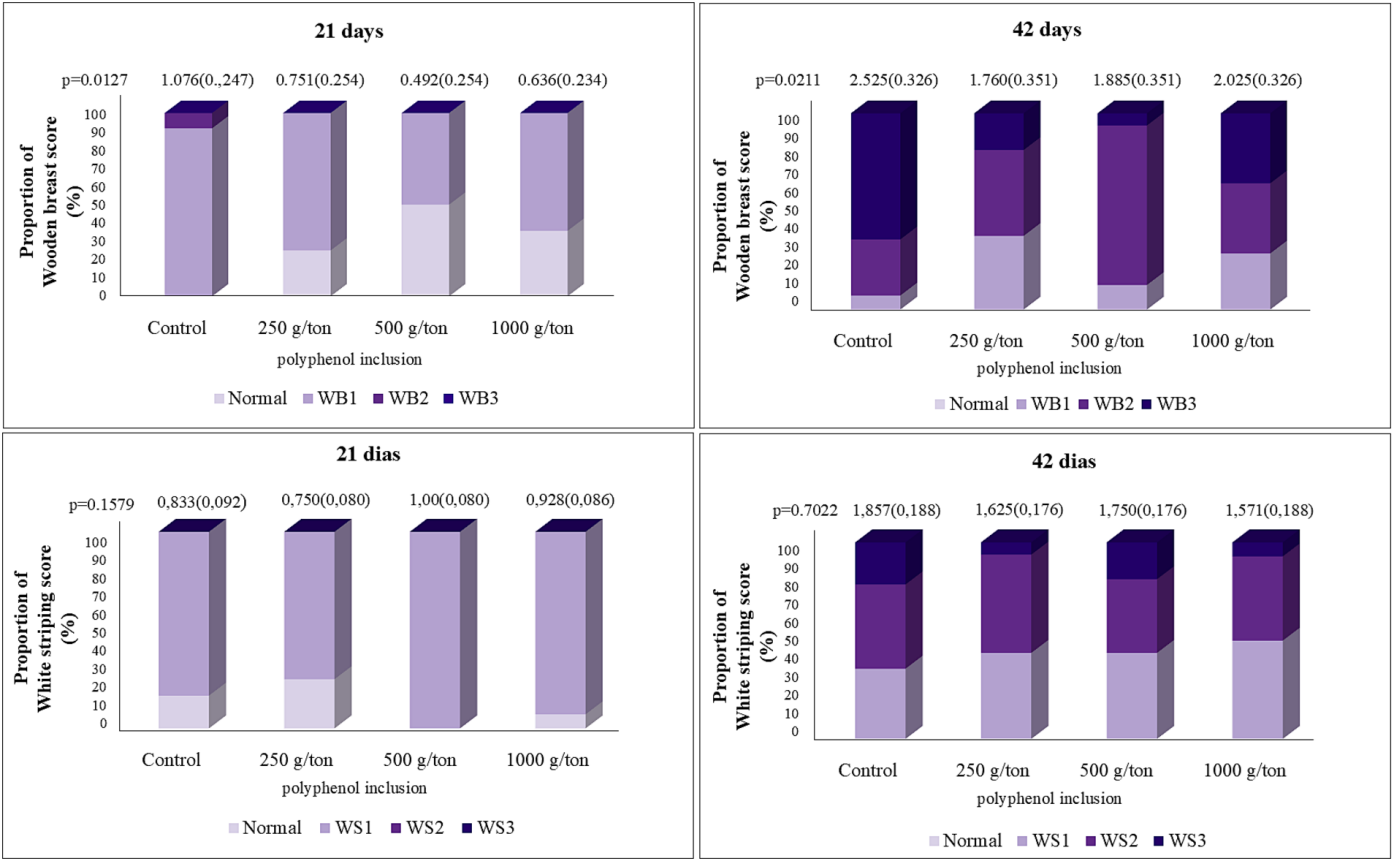
Quantification of pectoral myopathy scores in broiler chickens fed on different levels of polyphenol blend. Average estimated by the mathematical model. WB, wooden breast (21 days): Y = −0.00038X + 0.8967 a/b (SEM); WB (42 days): y = 0.0000009516×^2^ − 0.00126X + 1.8876 a/b (SEM).

At 21 days, wooden breast severity decreased linearly with increasing levels of the polyphenol blend (Figure 5). The polyphenol blend did not affect the incidence of white striping (data not shown).

At 42 days, wooden breast myopathy exhibited a quadratic trend, with the minimum severity estimated at 652 g/ton of polyphenol blend inclusion. No significant differences were observed for wooden breast severity at 28 and 35 days. No statistically significant differences were found for the evaluation of white striping.

Meat quality parameters evaluated at 21, 28, and 35 days were not influenced by the polyphenol blend (results not shown). However, at 42 days, cooking weight loss (CPP) decreased linearly as polyphenol inclusion increased (Table 5). Shear force exhibited a quadratic response, with the minimum shear force estimated at 609 g/ton of polyphenol blend inclusion.

**Table 5:** Meat quality parameters of broiler chickens slaughtered at 42 days of age fed on different levels of polyphenol blend.

Parameters		Blend of Polyphenols inclusion levels (g/ton)				SEM^1^	*p* ^2^
		0	250	500	1,000		
Filet weight^2^ (kg)		0.395	0.370	0.393	0.389	0.005	0.3943
Length (cm)		18.47	18.36	18.30	18.43	0.066	0.8141
Width (cm)		10.09	10.09	9.77	9.68	0.788	0.1426
Thickness (cm)		4.39	4.25	4.23	4.28	0.429	0.5235
pH		5.89	5.93	5.98	5.88	0.017	0.1147
Color	L^*^	49.99	50.12	49.50	49.50	0.826	0.0529
	a^*^	2.07	2.24	2.39	2.39	0.266	0.6364
	b^*^	6.30	6.55	6.18	6.18	0.676	0.5774
WRC^3^ (%)		33.70	32.20	33.00	32.67	0.388	0.5924
Drip loss (%)		4.01	3.53	3.74	3.12	0.124	0.0903
WLT^4^ (%)		4.09	3.73	3.09	3.37	0.195	0.3110
WLC^5^ (%)		23.41	20.99	21.50	19.09	0.516	0.0580
SF^6^ (kgf/cm^2^)		2.24	1.84	1.99	1.98	0.049	0.0406

^1^Standard error of the mean; ^2^Weight of cooled right filet; ^3^Water retention capacity; ^4^Weight loss due to thawing; ^5^Weight loss through cooking; ^6^Shear force; WRC: y = −0.00378x + 22.88951 r^2^ = 0.17 (*p* = 0.0008); SF: y = 0.0000007989x^2^-0.00097459x + 2.17653 r^2^ = 0.13 (*p* = 0.0440).

L*, a*, and b*: coordinates of the CIELAB color space, where L* represents lightness, a* the green–red axis, and b* the blue–yellow axis.

Regarding muscle tissue morphometry, at 21 days, fiber number per field showed a linear increase with increasing polyphenol inclusion, while fiber diameter and area decreased linearly (Table 6). At 42 days, the number of muscle fibers continued to increase linearly with polyphenol levels, while fiber area decreased. No significant differences in muscle morphometry were observed at 28 and 35 days of age.

**Table 6:** Morphometry of muscle fibers from the breast of broiler chickens fed on different levels of polyphenol blend.

Morphometry	Blend of polyphenols inclusion levels (g/ton)				SEM^1^	*p* value
	0	250	500	1,000		
21 days						
Number of fibers/field	110.14	119.46	133.54	148.67	3.25	<0.0010
Fiber diameter (μm)	67.28	65.95	61.99	59.12	0.92	0.0033
Fiber area (μm^2^)	2.385	2.322	2.174	1.790	57.11	0.0002
Area fibers/field (%)	70.46	41.41	72.70	72.21	0.60	0.6109
28 days						
Number of fibers/field	59.14	61.29	60.04	59.16	1.07	0.8869
Fiber diameter (μm)	93.20	92.51	88.88	91.71	0.92	0.3361
Fiber area (μm^2^)	3.930	3.933	4.112	3.962	107.45	0.9201
Area by fibers/field (%)	67.50	66.58	67.74	68.88	0.49	0.4490
35 days						
Number of fibers/field	46.00	45.41	45.08	45.09	0.67	0.9689
Fiber diameter (μm)	107.25	99.31	100.10	100.57	1.38	0.1639
Fiber area (μm^2^)	5.432	5.124	5.332	5.110	105.23	0.6815
Area by fibers/field (%)	61.50	62.59	65.52	63.14	0.67	0.1860
42 days						
Number of fibers/field	23.81	28.00	28.16	39.04	1.50	0.0011
Fiber diameter (μm)	124.47	118.82	115.55	116.62	2.26	0.5494
Fiber area (μm^2^)	7.906	7.203	6.987	5.535	258.74	0.0082
Area by fibers/field (%)	50.23	54.63	54.32	53.33	0.82	0.2238

^1^Standard error of the mean; 21 days: Number of fibers: Y = 0.03922x + 110.8614 r^2^ = 0.64 (*p* ≤ 0.0001); Fiber diameter (21 days): y = −0.0086x + 67.34455 r^2^ = 0.38 (*p* = 0.0004); fiber area: y = −0.61480x + 2439.92836 r^2^ = 0.51 (*p* < 0.0001); 42 days: number of fibers: y = 0.01464x + 23.29944 r^2^ = 0.64 (*p* = 0.0002); fiber area: y = −2.29274x + 7914.34994 r^2^: 0.39 (*p* = 0.0012).

### 3.3 Antioxidant status, lipid profile, and biochemical parameters

**Figure 6:**
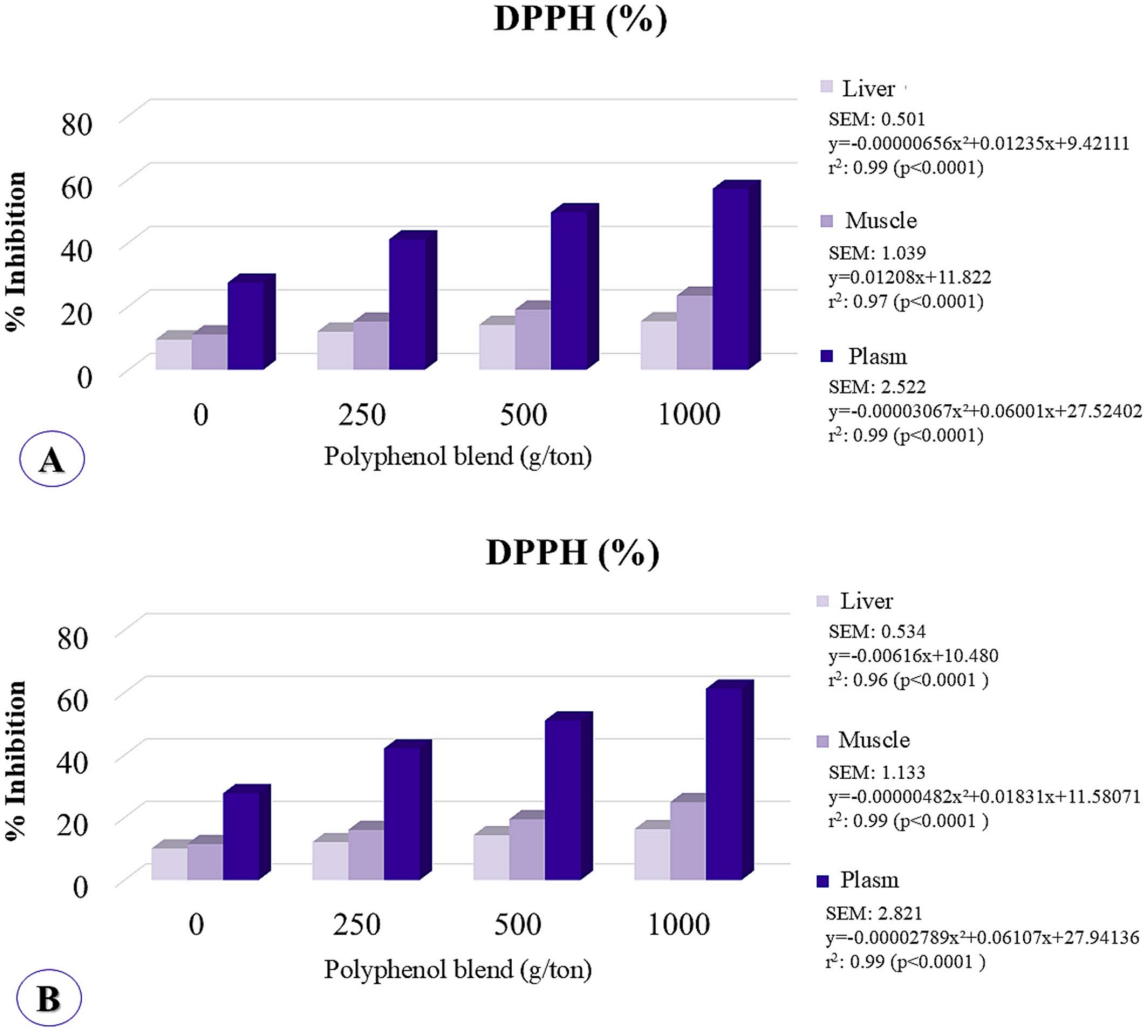
Hepatic, muscular, and serum concentrations of antioxidant potential were analyzed by the method of inhibiting DPPH (2,2-diphenyl-1-picrylhydrazyl) in broiler chickens fed on different levels of polyphenol inclusion. **(A)** Assessment at 21 days; **(B)** Assessment at 42 days.

Plasma antioxidant potential was significantly affected by polyphenol inclusion, with quadratic responses observed at 21 and 42 days. The maximum estimated inclusion levels were 978 g/ton at 21 days and beyond the tested range at 42 days (Figure 6).

In the breast muscle, DPPH inhibition demonstrated a linear increase at 21 days and exhibited quadratic behavior at 42 days, with the maximum point occurring beyond the tested range. In the liver, antioxidant potential followed a quadratic trend at 21 days, with the maximum inclusion level estimated at 941 g/ton, and displayed linear increasing behavior at 42 days. These findings indicate that higher levels of polyphenol inclusion enhance tissue antioxidant activity.

Capric acid (C10:0) and *α*-linolenic fatty acid (C18:3 ω3) showed a linear decrease with increasing levels of polyphenol blend inclusion. Conversely, palmitoleic acid (C16:1) exhibited a linear increase, indicating higher concentrations in the muscles of birds fed diets with greater polyphenol inclusion. Dihomo-*γ*-linolenic fatty acid (C20:3 ω6) followed a quadratic trend, with a maximum point estimated at 460 g/ton of polyphenol blend inclusion based on the regression equation (Table 7). Similarly, conjugated linoleic acid (CLA, C18:2) displayed quadratic behavior, with the optimal dosage estimated at 329 g/ton of polyphenols. Total omega-3 fatty acids exhibited a linear decrease with increasing polyphenol blend inclusion in the diet.

**Table 7:** Fatty acid profile of the breast muscle tissue (*Pectoralis major*) of broiler chickens fed with polyphenols and evaluated at 42 days of age.

Fatty acids^1^		Blend of polyphenols inclusion levels (g/ton)				SEM^2^	*p* ^3^
		0	250	500	1,000		
Capric	(C10:0)	0.110	0.106	0.100	0.100	0.001	<0.0001
Lauric	(C12:0)	0.208	0.208	0.206	0.204	0.001	0.6949
Mystic	(C14:0)	0.544	0.536	0.534	0.544	0.006	0.9173
Palmitic	(C16:0)	21.908	21.770	21.816	21.740	0.026	0.0932
Heptadecanoic	(C17:0)	0.358	0.354	0.360	0.354	0.002	0.8350
Stearic	(C18:0)	0.750	0.728	0.736	0.762	0.005	0.1616
Arachidic	(C20:0)	0.140	0.130	0.144	0.136	0.002	0.3170
Myristoleic	(C14:1)	0.192	0.202	0.186	0.196	0.002	0.1524
Palmitoleic	(C16:1)	1.644	1.676	1.756	1.734	0.012	0.0004
Oleic	(C18:1) ω9	28.138	28.306	28.727	28.262	0.034	0.3655
Linoleic	(C18:2) ω6	37.442	37.490	37.432	37.460	0.029	0.9202
α-linolenic	(C18:3) ω3	3.406	3.468	3.272	3.308	0.025	0.0088
Conjugated linoleic	(C18:2)	0.297	0.272	0.298	0.304	0.003	<0.0001
Dihomo-γ-linolenic	(C20:3) ω6	0.106	0.100	0.100	0.108	0.001	0.0036
Arachidonic	(C20:4) ω6	2.350	2.376	2.368	2.370	0.012	0.9086
Pentadecanoic	(C15:0)	0.152	0.150	0.144	0.148	0.001	0.5495
Eicosanoic	(C20:1)	0.314	0.316	0.318	0.320	0.003	0.9449
Eicosadienoic	(C20:2)	0.504	0.486	0.504	0.512	0.003	0.0622
Eicosatrienoic	(C20:3) ω3	1.300	1.300	1.314	1.298	0.005	0.7955
Eicosapentaenoic	(C20:5) ω3	0.144	0.136	0.140	0.140	0.001	0.5014
Totally saturated		24.170	23.872	24.040	23.988	0.043	0.0909
Totally unsaturated		75.646	76.254	76.036	75.980	0.043	0.0909
Monounsaturated		30.288	30.500	30.532	30.512	0.04	0.0956
Polyunsaturated		45.542	45.628	45.428	45.500	0.038	0.3290
Total ω3		4.850	4.904	4.726	4.746	0.023	0.0097
Total ω6		40.692	40.724	40.702	40.754	0.032	0.9257
Total ω9		30.096	30.298	30.346	30.316	0.039	0.0949
Unsaturated/saturated		3.137	3.189	3.160	3.168	0.007	0.0940
Polyunsaturated/saturated		1.884	1.911	1.889	1.896	0.004	0.1536
ω6/ω3		8.392	8.308	8.613	8.589	0.042	0.1310

^1^Fatty acids are presented in percentages calculated regarding the quantification of total fatty acids. ^2^Standard error of the mean; ^3^Results were statistically significant at *p* < 0.05. Capric acid y = −0.00001006x + 0.10840 r^2^0.57 (*p* < 0.0001); Palmitoleic acid y = 0.00009440x + 1.6612 r^2^0.40 (*p* = 0.0001); α-linolenic acid y = −0.00013737 + 3.42360 r^2^0.21 (*p* = 0.0015); Conjugated linoleic acid y = 0.00000004404454x^2^ − 0.00002906x + 0.2908 r^2^0.32 (*p* = 0.0002); Dihomo-γ-linolenic acid y = 0.00000002981818x^2^ − 0.00002745x + 0.10573 r^2^0.55 (*p* = 0.0004); Total ω3 y = −0.00013966x + 4.8676 r^2^0.25 (*p* = 0.0087).

No significant differences were observed in blood biochemical parameters, including alanine aminotransferase (ALT), aspartate aminotransferase (AST), and lactate levels (data not shown).

## 4 Discussion

The dietary inclusion of the polyphenol blend did not affect weight gain or feed intake during the pre-starter phase. However, broiler chicks supplemented with the polyphenol blend showed significant improvements in all performance parameters during the starter and growing phases, as well as over the entire period, indicating more efficient nutrient utilization. This finding contrasts with a study by Jamroz et al. (39), which found no significant effects on body weight gain or feed conversion when chickens were supplemented with a tannin compound from Chestnut (*Castanea sativa*) at concentrations of 250 or 500 mg/kg. Additionally, Zhou et al. (40) demonstrated that the positive effects of flavonoids are dose-dependent. These varying results suggest that the benefits of polyphenols depend on factors such as the specific type of polyphenol, the effective concentrations of bioactive molecules reaching the epithelium, and the metabolites produced by the gut microbiota, which may influence performance. Therefore, the correct dosage of polyphenols in feed is crucial for producing beneficial effects in chickens, and this also depends on the standardization of the plant extract.

The inclusion of the polyphenol blend in the diet from 1 to 42 days of age resulted in favorable production parameters. The optimal inclusion levels were estimated to be between 514 and 664 g/ton for weight gain and productive efficiency index, respectively. Current research supports the idea that appropriate amounts of polyphenols, such as tannic acid in the range of 0.5 g/kg to 5 g/kg, can improve performance and intestinal health due to their potential antimicrobial, antioxidant, and anti-inflammatory effects (41–44). However, higher doses of flavonoids may have adverse effects on performance (40).

Importantly, polyphenol blend supplementation influenced the growth pattern of various body parts in broiler chickens (Table 3). At 500 g/ton, there was a delay in breast growth compared to leg growth. However, no difference was observed in breast yield at 42 days of age (Table 2), suggesting that there was no reduction in protein deposition in the breast muscle, but rather an adjustment in the timing of growth for different body parts. This may also be related to the findings on Wooden breast myopathy, where an inclusion level of 654 g/ton led to a reduction in the severity of this myopathy. The results observed in the growth and development of muscle tissue can be explained by the myoprotective properties attributed to polyphenolic compounds, including: (1) enhancement of mitochondrial function and biogenesis; (2) induction of antioxidant enzymes and protection against mitochondrial dysfunction; (3) promotion of muscle angiogenesis, energy capacity, and contractile function; and (4) stimulation of protein synthesis, myotube differentiation, and hypertrophy of muscle fibers (45–47). While the exact biochemical mechanisms through which polyphenolic compounds protect skeletal muscle cells are not fully understood (48), their protective actions may occur at the intracellular level, through direct or indirect interactions with transcription factors such as peroxisome proliferator-activated receptor gamma coactivator 1-alpha (PGC-1α), Nuclear Respiratory Factor 1 (Nrf1), mitochondrial transcription factor A (TFAM), or myogenic regulators such as myogenin, Myogenic Factor 5 (Myf5), and Myoblast Determination Protein 1 (MyoD) (49).

The polyphenol blend used in this study contains different plant extracts, and its effects may arise from the combination of various molecules, such as condensed hydrolyzable tannins and flavonoids. Notably, ellagitannins, a class of hydrolyzable tannins, release a sugar molecule and several gallic acid and ellagic acid molecules upon hydrolysis (50). Gallic acid is easily absorbed into the bloodstream (51), while ellagic acid has low bioavailability due to its strong affinity for proteins and poor absorption (52). However, as Espín et al. (53) noted, ellagitannins undergo hydrolysis in the jejunum, increasing the amounts of ellagic acid and its derivatives, which are then metabolized by the intestinal microbiota. This metabolism leads to the production of compounds such as urolithins, which enhance cellular health and promote mitophagy and mitochondrial function, in addition to combating disorders related to muscles and the brain (54). Furthermore, intestinal metabolites of ellagic acid can produce urolithin A or urolithin B (55), both of which have myoprotective effects (56–58). Urolithin A has been shown to improve muscle strength and endurance (56, 57). The biological functions of bioactive compounds depend on their metabolites produced through intestinal and hepatic metabolism (59), highlighting the importance of intestinal health in the utilization of these compounds. To benefit from the muscle-related effects of ellagitannin supplements, individuals must possess an intestinal microbiome that includes representative amounts of *Akkermansia muciniphila* and *Lactobacillus* spp. (60, 61). *Akkermansia muciniphila* is associated with muscle development (62), while Lactobacillus spp. is linked to increased muscle mass and strength (63). Given these findings, further research should investigate the effects of polyphenol blend administration on the intestinal microbiome.

Studies have demonstrated that urolithin B has an androgenic effect on skeletal muscle without negatively impacting the development of other body tissues (58, 64). *In vitro* studies further show that urolithin B stimulates protein synthesis while reducing protein degradation (58). Additionally, this metabolite of ellagic acid exhibits anti-inflammatory effects by decreasing the expression of several cytokines, including interleukin-6 (IL-6), interferon-*γ* (IFN-γ), tumor necrosis factor-*α* (TNF-α), interleukin-4 (IL-4), and interleukin-1β (IL-1β) (65). Xing et al. (66) found that IL-6, IL-1β, and TNF-α mRNA expressions were elevated in muscles affected by Wooden breast myopathy. For instance, TNF-α contributes to muscle loss by inhibiting muscle regeneration, maintaining satellite cells in the proliferation stage and preventing their differentiation (67). The presence of IL-1β acts as a predisposing factor for impaired muscle regeneration, and its inhibition promotes muscle differentiation (68). Therefore, modulating the expression of inflammatory cytokines can improve inflammatory tissue damage and facilitate the regenerative phase of muscle affected by Wooden breast.

Furthermore, ellagitannins and ellagic acid possess phenolic hydroxyl groups, which contribute to the stabilization of reactive oxygen species (ROS) due to their ability to donate H+ radicals, thus remaining stable. As a result, they exhibit potent and sustained antioxidant effects both *in vitro* and *in vivo*, providing significant protection against ROS (69–71). Polyphenols can selectively induce the expression of antioxidant enzymes through the modulation of redox-sensitive signaling pathways, inhibiting lipid peroxidation, neutralizing free radicals (56, 72), and influencing muscle development as well as pectoral myopathies. Moreover, oxidative stress plays a pathogenic role in inflammatory diseases (73), including muscle conditions such as White striping and Wooden breast. Thus, evaluating the effects of polyphenols on the antioxidant capacity of broiler chickens is critical. As previously hypothesized, this study suggests that the polyphenol blend could positively impact the reduction of pectoral myopathies by modulating oxidative stress. At the molecular level, gene expression and metabolomics studies confirm that oxidative stress is a key factor in the development of pectoral myopathies (8, 13, 74). Accordingly, the use of a polyphenol blend in the diet positively influenced the antioxidant potential of plasma, muscle, and liver in chickens, as evaluated using the DPPH inhibition method (Figure 5). The DPPH radical is a widely used indicator for measuring antioxidant potential in various tissues and substances, including plant extracts, chemical compounds, and foods (75). It is employed to assess the capacity to neutralize free radicals, which are strongly associated with cellular aging and tissue damage (76). Polyphenols possess strong antioxidant properties due to the presence of one or more aromatic rings with hydroxyl groups that can interact with free radicals to form phenoxyl radicals, a structure responsible for their antioxidant activity (77–79). Therefore, the polyphenol blend may also mitigate lipid oxidation by inhibiting the formation of reactive oxygen species and acting as metal ion chelators, which catalyze oxidation reactions (80).

The morphometry of the pectoral muscle tissue further supports the reduction in the severity of myopathies. It reveals that higher levels of polyphenol blend inclusion lead to smaller muscle fiber areas, compensating for this reduction in fiber diameter with an increased fiber count per unit area. These findings explain why there were no losses in carcass and breast yield in birds at 42 days of age. This is likely due to the myoprotective action of compounds derived from the hydrolysis of previously described components. Therefore, the factors discussed may be correlated with the development of the pectoral muscle, with improvements in meat tenderness and a reduction in cooking-induced weight loss. A significant decrease in protein content and an increase in collagen in muscles affected by Wooden breast myopathy have been widely reported. These changes are likely related to progressive myodegeneration, deposition of interstitial connective tissue, and fibrosis (1, 14, 81). This finding corroborates several studies that have shown that severe Wooden breast increases meat toughness, drip loss, and moisture loss during cooking (82–85). Furthermore, relatively large muscle fibers are responsible for the coarse and undesirable texture of cross-cut meat (86).

When considering the quality of chicken products, fatty acids also play a significant role, as the use of natural food additives can alter the lipid profile of intramuscular fat (87) and directly impact meat quality and organoleptic properties. The administration of polyphenols has been shown to modify the lipid composition of animal products (88, 89). The analysis in this study demonstrated an effect on specific fatty acids, such as palmitoleic acid (C16:1), *α*-linolenic acid (C18:3), conjugated linoleic acid (C18:2), and dihomo-*γ*-linolenic acid (C20:3). Furthermore, a decrease in omega-3 fatty acid concentrations was observed as the inclusion levels of the polyphenol blend increased (Table 7). These variations may be attributed to the interaction between the metabolic processes involved in the synthesis of these fatty acids and the compounds in the polyphenol extract, as well as their capacity to influence lipid absorption. However, the reduction of omega-3 fatty acids may contribute to an extended shelf life for meat products by decreasing the presence of long-chain fatty acids, which are more prone to oxidation. These findings suggest that further studies on metabolic pathways could provide a deeper understanding of the effects of these compounds on the animal organism. Tannins and flavonoids are known to influence lipid metabolism in various ways. For example, tannins can reduce lipid absorption in the intestine by forming tannin-lipid complexes (90), thereby altering the physical properties of lipids and potentially affecting their bioavailability and functionality. The hypotriglyceridemic effect of proanthocyanidins found in chestnut wood, for example, is associated with reduced lipid absorption by enterocytes, leading to increased fecal excretion of cholesterol (91). Moreover, polyphenolic compounds can modulate lipid metabolism by influencing gene expression and enzymatic activity involved in lipogenesis, lipolysis, and cholesterol synthesis (92, 93). Another hypothesis is that bioactive compounds may directly influence the intestinal microbiota, which in turn could modulate the absorption, bioavailability, and biotransformation of fatty acids (94). Certain microbial species, such as Bacillus proteus or *Lactobacillus plantarum*, have been shown to convert *α*-linolenic acid (C18:3n-3), a precursor of omega-3 polyunsaturated fatty acids (PUFAs), into conjugated linoleic acid (C18:2), which is then further hydrogenated to saturated fatty acids like stearic acid (C18:0), thereby reducing the composition of omega-3 PUFAs (95).

Based on what has been explained so far, polyphenol blend reduced the incidence of Wooden Breast myopathy at both 21 and 42 days of age, likely due to its antioxidant potential, as observed in various tissues, and the modulation of muscle fiber size in the breast. This is associated with improved meat tenderness and reduced weight loss during cooking. Additionally, in birds fed with 500 g/ton of polyphenol blend, breast growth was delayed compared to other body parts, suggesting a modulation of allometric growth.

Supplementation with the polyphenol blend also improved the animals’ zootechnical performance, including feed consumption, weight gain, and feed conversion. The best value for the European productive efficiency index was observed at a dose of 514 g/ton of extract at 42 days of age.

## Data Availability

The raw data supporting the conclusions of this article will be made available by the authors, without undue reservation.
